# Perspectives of Local Community Leaders, Health Care Workers, Volunteers, Policy Makers and Academia on Climate Change Related Health Risks in Mukuru Informal Settlement in Nairobi, Kenya—A Qualitative Study

**DOI:** 10.3390/ijerph182212241

**Published:** 2021-11-22

**Authors:** Johanne Greibe Andersen, Catherine Karekezi, Zipporah Ali, Gerald Yonga, Per Kallestrup, Christian Kraef

**Affiliations:** 1 Center for Global Health, Department of Public Health, Aarhus University, 8000 Aarhus, Denmark; per.kallestrup@ph.au.dk (P.K.); christiankraef@googlemail.com (C.K.); 2 Danish Non-Communicable Diseases Alliance, 2100 Copenhagen, Denmark; 3 Kenya Diabetes Management and Information Centre, Nairobi 00100, Kenya; catherinekarekezi@yahoo.co.uk; 4 Non-Communicable Diseases Alliance of Kenya, Nairobi 00100, Kenya; zippy.ali@gmail.com (Z.A.); yongag@gmail.com (G.Y.); 5 School of Medicine, University of Nairobi, Nairobi 00100, Kenya; 6 Heidelberg Institute of Global Health, University of Heidelberg, 69117 Heidelberg, Germany

**Keywords:** climate change, environmental health, informal settlements, slums, non-communicable diseases, communicable diseases, low- and middle-income countries

## Abstract

Sub-Saharan Africa has been identified as one of the most vulnerable regions to climate change. The objective of this study was to explore knowledge and perspectives on climate change and health-related issues, with a particular focus on non-communicable diseases, in the informal settlement (urban slum) of Mukuru in Nairobi, Kenya. Three focus group discussions and five in-depth interviews were conducted with total of 28 participants representing local community leaders, health care workers, volunteers, policy makers and academia. Data were collected using semi-structured interview guides and analyzed using grounded theory. Seven main themes emerged: climate change related diseases, nutrition and access to clean water, environmental risk factors, urban planning and public infrastructure, economic risk factors, vulnerable groups, and adaptation strategies. All participants were conscious of a link between climate change and health. This is the first qualitative study on climate change and health in an informal settlement in Africa. The study provides important information on perceived health risks, risk factors and adaptation strategies related to climate change. This can inform policy making, urban planning and health care, and guide future research. One important strategy to adapt to climate change-associated health risks is to provide training of local communities, thus ensuring adaptation strategies and climate change advocacy.

## 1. Introduction

According to the United Nations (UN) Intergovernmental Panel on Climate Change (IPCC), climate change is one of the current biggest global challenges [1,2]. The UN defines climate change such as extreme weather events as attributed directly or indirectly to human activity [1,2]. Climate change has also impacted the World Health Organization’s (WHO) list of urgent health challenges for the decade leading up to the deadline of the Sustainable Development Goals (SDGs) in 2030 [3,4]. Concomitantly, non-communicable diseases (NCDs), defined by WHO as chronic diseases that are not passed from person to person, are the fastest growing global health burden. Both crises are interlinked and require immediate preventive measures [5]. Climate change will directly and indirectly increase NCD-related morbidity, disability and mortality [5]. The climate change effects are both direct, with extreme weather events such as droughts, floods, landslides, and sea level rises, and indirect, including poor water quality and malnutrition. Climate change is likely to cause an increase in communicable diseases (CDs) in addition to NCDs such as mental illness, malnutrition, allergies, cardiovascular diseases, injuries, respiratory diseases and poisoning according to Watts et al. [6,7,8].

Climate change and many NCDs have the same root causes. The most obvious cause is air pollution, which causes an estimated 7 million deaths annually [3]. Changing weather conditions also lead to agricultural insecurity and changed food systems. A dietary transition from locally sourced, unprocessed foods, to imported, over-processed foods and industrial dairy and meat production, increases the risk for hypertension and obesity, causing up to 15 million deaths annually. These linkages between climate change and NCDs highlight the need for multi-sectoral approaches and development of policies and practices to ensure adaptative measures and improve resilience to climate change.

Sub-Saharan Africa has been identified as one of the most vulnerable regions to the impact of climate change. In addition, health systems in the region are insufficiently equipped to handle the increase in climate-related morbidity. The prevalence of NCDs is increasing rapidly in urban areas in East Africa. According to WHO, the Africa region will experience a 27% increase (compared to 17% globally) in additional deaths due to NCDs during the next decade [5]. This will result in 28 million additional deaths in the region and thus a considerable disease burden [5]. Climate change will further increase morbidity and mortality in people with NCDs, in particular hypertension, obesity and asthma.

An estimated 1 billion people live in slum areas worldwide [9]. Slum households and informal settlements are defined by the UN-Habitat as “*One in which inhabitants suffer one or more of the following ‘household deprivations’: lack of access to improved water source, lack of access to improved sanitation facilities, lack of sufficient living area, lack of housing durability and lack of secure tenure*” [10], “*Urban regulations developed outside the formal system recording land ownership, land tenure and a range of regulations relating to planning and land use, built structures and health and safety*” [11]. Poor housing, lack of basic infrastructure and health challenges make inhabitants of informal settlements especially vulnerable to the health effects of climate change [12]. Residents of informal settlements are often neglected in research, and health-related data and information are thus very limited [13,14].

In 2016, the Mukuru informal settlement (Kwa Njenga, Kwa Reuben and Viwandani) was estimated to have 301,683 inhabitants, living in 100,562 households [15]. Conservative population projections of a six percent annual growth rate estimate that, in 2030, Mukuru will have 682,076 inhabitants [15]. At present, the informal settlement Mukuru in Nairobi, Kenya, already experiences significant environmental risks that will likely be exacerbated by climate change and significantly increase the vulnerability to displacement, disease and risk of premature death [15]. A number of air pollution sources make the air in Mukuru unsafe [15]. A previous study has shown that 19% of individuals in Mukuru experienced coughing in the previous six months and 43% of households reported coughing as a top reason for a household member to visit a health facility in the previous six months [15]. The Ngong River, which runs through Mukuru, is to a large extent exposed to industrial waste, which contributes to toxic pollution. Mukuru’s proximity to the Ngong River, its lower elevation than the average elevation in Nairobi and black clay soil, combined with many unpaved roads, narrow footpaths and poor waste management are all factors contributing to increased flood risk and frequent flooding in Mukuru. The area experiences a series of flood events, especially during the two rainy seasons in March–May and October–December [16]. From 2002 to 2010, the Ngong River riparian zone has changed from being largely undeveloped to completely developed, thus contributing to more severe flooding in conjunction with climate change-induced extreme weather events [15]. In 2018, 27% of households in a survey in Mukuru reported experiencing flooding in the six months prior to the survey. In 2016, 57% of the households reporting flooding in the six months preceding the survey and were impacted by health issues, compared to just 40% of households that did not flood [16]. In 2016, 50% of residents in Mukuru experienced not having enough food at least once, compared with 30% of residents in formal settlements in Nairobi. Furthermore, 12% in Mukuru were without food most days [15]. Food insecurity and malnutrition are likely to be exacerbated by climate change [15]. Mukuru is very far from meeting the SDGs, particularly those related to poverty, water, sanitation, electricity and gender. Air pollution and extreme weather, floods, droughts and humidity affect access to healthy diets, physical activity and mental health in informal settlements such as Mukuru [15]. In the future, Mukuru will likely experience more flooding, heat waves, drought-induced drinking water scarcity, sinking land, and food and water insecurity due to climate and related weather events [15]. The aim is to build resilience of the poor and those in vulnerable situations, and reduce their exposure to climate-related extreme events by 2030 [15].

Evidence on the knowledge, attitudes and practices regarding climate change adaptation and resilience is very limited. There is a scarcity of data regarding the perceived relationship between climate change and related NCDs in low- and middle-income countries (LMICs). In the future, both climate change hazards and NCDs will affect a growing number of residents in informal settlements with an increasing population density. Assessing and mapping knowledge, attitudes and practices regarding both direct and indirect effects of climate change related health threats is crucial to guide future interventions on climate change adaptation and resilience in high-density informal settlements such as Mukuru in East Africa and the world.

The objective of this study was to explore the knowledge base on climate change related health risks and adaptation strategies among local formal and informal community leaders, health care workers and volunteers, and key informants working in academia and policy on climate change in Nairobi, Kenya.

## 2. Materials and Methods

### 2.1. Study Design and Setting

The study was conducted among 28 people working with climate change related health issues related to the Mukuru informal settlement in Nairobi, Kenya. Mukuru is one of the largest informal settlements and was selected as the study setting. Mukuru is one of the fastest growing informal settlements in East Africa, and is surrounded by major industrial complexes, which contribute to environmental health risks closely linked to climate change (e.g., air pollution, toxic wastewater and sealing of surfaces that contribute to flooding). In Mukuru, 92% of residents are renters/tenants and housing usually consists of 10 × 10 feet structures with walls and roof made of sheet metal, frequently with dirt floors [15]. The perspectives of formal and community leaders, health workers and volunteers working in the informal settlement of Mukuru, Nairobi, Kenya were obtained in five in-depth interviews (IDI) and three focus group discussions (FGDs).

### 2.2. Participants and Sampling

In total, 28 participants (13 male and 15 female) took part in this study; three FGDs were conducted with 23 participants, in addition to five IDIs with key informants. Participants in FGDs were invited using convenience sampling from three purposively selected groups; Health Care Professionals (HCPs) (*n* = 6), Community Health Volunteers (CHVs) (*n* = 7) and local community leaders (e.g., mobilizers, chairpersons, opinion leaders) (*n* = 10). The five purposively sampled informants for IDIs were recruited through their workplaces—African Population and Health Research Centre, Stockholm Environmental Institute Africa, Kenyatta University, and the Ministry of Health, Kenya. Participants were also sampled from among the NCD Alliance of Kenya (NCDAK) network. All participants were first contacted by email by the staff of NCDAK, a non-governmental, civil-society organization coordinating multi-stakeholder activities on NCDs.

### 2.3. Data Collection

Data were collected from March to May 2021. Three FGDs with 6–10 participants each were conducted. Each FGD session lasted 80–120 min and took place in a centrally located conference room in Nairobi. Five IDIs each lasting 35–50 min were held at the informant’s workplace. Due to COVID-19 restrictions, one FGD and two IDIs had to be conducted online. Semi-structured questionnaires with open-ended and probing questions were used for the FGDs and IDIs. To ensure validity, the questionnaire guides were constructed congruently with the specific research questions based on guides from similar studies identified by a thorough literature review, and modified to the Kenyan context [6,17,18]. The questionnaires were developed in English, evaluated by two independent researchers (Johanne Greibe Andersen and Christian Kraef) in addition, NCDAK evaluated and pilot tested the tools to confirm their validity. A trained moderator guided the interviews and an assistant took notes. Two NCDAK staff members were also present during the interviews. The moderator identified when data saturation had been reached. All interviews were conducted in English and, when necessary, explained in Swahili or Sheng, a mixed language commonly used among poorer people in Nairobi. Interviews were audio-recorded and transcribed verbatim, and non-English phrases were translated into English. Transcripts were not returned to the participants for comments.

### 2.4. Data Analysis

A grounded theory approach with open coding was used to inductively construct coding categories and concepts to identify key themes emerging from the data [19]. Before coding, interview transcripts were read numerous times to become familiar with the discussions and meanings. The data and codes were reviewed by two researchers (Johanne Greibe Andersen and Christian Kraef) using the computer software program NVivo (QSR International, Doncaster, Australia). A thematic analysis was conducted to enhance reliability. Using an inductive approach, key themes were identified to create coding categories for data analysis. Codes were merged, and subcategories and hierarchical coding structures emerged along with key themes.

### 2.5. Ethics

The study objectives were made clear to all the participants, and that participation in the study was voluntary and anonymous. All participants provided written informed consent before the interviews. The audio files and transcripts were stored safely and were accessible only to the investigators. This study received ethical approval from Amref Ethics and Scientific Review Committee (ESRC) with the protocol number P906/2020.

The data collection process respected local restrictions and regulations regarding COVID-19, e.g., by providing hand sanitizer and face masks, ensuring physical distance by limiting the number of people in a room, and observing curfews.

## 3. Results

The data analysis resulted in seven main themes: climate change related diseases, nutrition and access to clean water, environmental risk factors, urban planning and public infrastructure, economic factors, vulnerable groups, and adaptation strategies. The results are presented according to themes and sub-themes (Table 1). A representation of sub-themes across the FGDs and IDIs is listed in Table 2.

### 3.1. Climate Change Related Diseases

A wide variety of climate change related NCDs were mentioned in the eight interviews—hypertension, heart attack, lung disease, diabetes, cancer, kidney disease, heat stroke, ulcer, sunburn, and obesity. Mental stress related to climate change was mentioned in most interviews, “*the fact that you’re not able to anticipate, to plan, because it is not known what will happen tomorrow, that psychological stress also strains the body and I think it also has an effect*” (IDI 3). Injuries and accidents related to both traffic and flooding were also highlighted as adverse effects related to climate change. Finally, infectious diseases were mentioned by the informants, such as waterborne diseases (cholera, typhoid, diarrhea), malaria, respiratory tract infections (including tuberculosis), seasonal flu, and COVID-19- Climate change, “*will cause people to get cholera. Especially when the floods come, our pipes, the water which comes from the tap, is connected with the sewer line*” (FGD, CHV), and, “*many people get malaria as a result of the water which has been stagnant due to flooding*” (FGD, Community Leader).

### 3.2. Nutrition and Access to Clean Water

Food insecurity and rising food prices were mentioned as main challenges caused by climate change in all of the eight interviews (all FGDs and IDIs), “*The issue of food security of course it’s also a problem because when we have harsh weather patterns, it also affects agricultural production, and you see, because in urban areas we don’t farm so they rely on food to be transported from other areas. Of course, the cost of that food will go up in the urban areas. It’s a challenge*” (IDI 2). Moreover, malnutrition in relation to climate change was mentioned as a threat, “*If there’s crop failure due to drought, it means people will not get food*” (IDI 1). “*There’s no food and you used to feed your children so well. They used to eat greens, protein, starch, things like that and now it is so hot. Everything is gone*” (FGD, Community Leader). Poor quality of food and lack of access to good quality food was also mentioned as an important issue, “*Now people are forced to use more chemicals in the food production chain*” (IDI 5). The impacts of the available and affordable food on health were noted: “*Actually, we see now the canned food, the processed food is more easily available than the natural food, so you get (…) more starchy foods, and which is not healthy so diabetes comes in*” (FGD, HCP). Further, rising food prices was a major issue, “*The prices of fish is really high. Fish from a fresh water lake has been a challenge*” (FGD, HCP). Insecure water supplies are largely affected by extreme weather such as droughts or seasonal changes. This was reported to cause distress and disease in the informal settlement, “*When there is lack of water and most of the taps are dry, these illegal water vendors come in with polluted water, dirty water*” (FGD, Community Leader). “*When drought occurs, when we have prolonged drought seasons, that affects our water resources that eventually now lead to water scarcity*” (IDI 3).

### 3.3. Environmental Risk Factors

Air pollution was regarded as a major issue in the informal settlement across all interviews, “*There’s a lot of air pollution both indoor and outdoor*” (IDI 5). “*We are surrounded by a lot of industries, the way they dispose their waste changes the climate, and also the pollution, air pollution, within Mukuru*” (FGD, CHV). “*We have seen a high number in cases of NCDs. Also due to pollution of air around Mukuru, we’ve seen children developing asthma at a very young age and even adults are coming in with asthma because of air pollution caused by the factories and industries and all that*” (FGD, HCP). Indoor air pollution was also regarded as an issue for NCDs in the informal settlements, “*Indoor pollution is also a challenge. They either use charcoal or they use paraffin. (…) What they inhale, it’s also a challenge if you look at some of the respiratory illnesses*” (IDI 2). Furthermore, many respondents mentioned contamination of food and water, “*When I see those sukuma wiki (kale type of vegetable) they look very clean, very nice, but you know they are already contaminated because the water they use is that sewerage (water)*” (FGD, Community Leader), and “*There are companies emitting mercury, lead, zinc and some other heavy metals which is a recipe for some dangerous diseases, dangerous NCDs like cancer and so on*” (FGD, Community Leader). “*The industries, they are urged to avoid flooding the rivers with bio-hazardous chemicals*” (IDI 5). All respondent groups also brought up a lack of proper waste management as a pressing issue, “*In Mukuru, we don’t have exactly a place to put our dirty waste. So, everybody will just throw their waste wherever they wish*” (FGD, Community Leader).

### 3.4. Urban Planning and Public Infrastructure 

Infrastructure and urban planning, in general, were additional main themes across the interviews, “*There is no proper arrangement of how those buildings are being constructed. Secondly, there is no right sewer lines. There are no right drains. There are no proper roads*” (FGD, Community Leader). “*One immediate thing when you look at our mobility patterns, when we move from point A to point B, of course, if it rains, it increases your travel time, so a distance where you could be taking 45 min (…), you find it is taking you 1.5 h or 2 h to reach your destination*” (IDI 2). Public transportation costs were mentioned to triple during rainy seasons. 

Poor housing and vulnerable placements of housing were a major concern for many informants, “*The houses near the stream or next to water sources are not expensive, they are cheap*” (FGD, CHV). “*Earlier I never heard about landslides but now it is something that is happening now and then. People are being told: don’t live in this area because when it rains this area, it is sliding*” (FGD, Community Leader). Migration and displacement of people due to climate change, e.g., losing their homes, was mentioned, “*When there’s flooding, the people who live in that area are forced to vacate*” (FGD, CHV). This was also stated to be a problem, e.g., for following up in immunization programs, “*we can’t immunize (them), maybe they moved away to search for another place and they are not traceable*” (IDI 5). Furthermore, it was stated that the local health services are not always climate change resilient, “*we’ve had some of our facilities ruined because of the weather (…) because of either floodings…*” (IDI 5). This further negatively impacts the ability to detect and treat NCDs, “*there is no community structures, there’s no healthcare facilities, so you’re not able to catch these people early enough, so they’ll easily progress to get non-communicable diseases*” (IDI 5). 

Lack of proper sewage and drainage systems in the informal settlement were highlighted, and the need to encourage authorities to ensure better sewage, was brought up, “*They should build manholes for us, so that when the sewage burst it will flow into the manhole instead of into people’s houses*” (FGD, CHV). “*The flooding incidences, they really also affect our quality of life especially in these residences particularly the informal settlements because there’s no infrastructure, the storm water drains are not there, so eventually what we have is the waterborne diseases because they probably will have a mix-up of storm water into the drinking water and of course sanitation will also be affected because if you look at the type of facilities that they use, they are likely to be affected by that*” (IDI 3).

Finally, deforestation as part of climate change related hazards, in addition to the need to replant trees, was mentioned on multiple occasions during the interviews, “*The country is warming up because people are clearing vegetation and erecting high-rise buildings which tend to interfere with the ecosystem and the weather patterns keep on changing based on that*” (IDI 1).

### 3.5. Economic Risk Factors

Generally, poverty related risk factors were mentioned in all interviews as a main factor for climate change related health hazards, “*Access to health services for those who fall sick, especially in the informal settlements due to climate change, is not something that they can easily get because some of them-if you look at their economic status-cannot afford the quality healthcare in high end institutions, health institutions, and if they access healthcare in the local, let’s say, county hospitals, most of the times they don’t have the capacity to handle such cases*” (IDI 1). Lack of insurance against climate change hazards in Kenya and Mukuru was also mentioned.

### 3.6. Vulnerable Groups

Poverty in general makes many residents in informal settlements vulnerable when natural disasters occur, “*The vulnerable people, they don’t have anywhere to go so they don’t. (…) they can’t go anywhere because they don’t have any other place they can go to. They end up being homeless*” (FGD, CHV). Children were stated as a vulnerable group in many interviews, “*Especially the children are affected when the floods are all over our place. Because these kids don’t know if water whether it’s good or bad or whether it’s from the sewer line. They will just play with it and that’s where they get these diseases, the waterborne disease*” (FGD, Community Leader). The elderly were also mentioned as another vulnerable group in the informal settlements, “*The air is polluted, like maybe industries emitting the fumes, maybe garbage being burnt somewhere, you see, the old suffer a lot because their respiratory organs are a little bit weaker*” (FGD, Community Leader). People living with NCDs (PLWNCD) were mentioned as vulnerable by several respondents, “*Because they are suffering from a non-communicable disease (…) they are vulnerable and some of the impacts of climate change would actually be a risk to them*” (IDI 4).

### 3.7. Adaptation Strategies

Respondents also offered insights into potential adaptation strategies. In terms of urban planning, it was suggested “*Let’s transform our cities from highly motorable cities to walking cities. From a planning perspective, I see a core benefit with NCDs because this will provide room for exercise, room for clean air in areas where we have for recreation, and this will be good for our health*” (IDI 4). Furthermore, reforestation and planting of trees and vegetation were brought up by many respondents.

In all eight interviews (FGDs and IDIs) it was stressed that educational measures must be taken to empower the residents in the informal settlements, and a willingness to learn and disseminate knowledge on climate change was emphasized, “*Train us! I think we should be having more knowledge on how to help ourselves if the climate changes*” (FGD, CHV). “*By creating awareness, it’s going to change the attitudes and perceptions of the community on climate change and its impacts*” (IDI 4). Regarding health and NCDs, it was mentioned, “*Also, we’ll have more people getting non-communicable diseases because the opportunity for educating the public on how to prevent non-communicable diseases, which is done both at community and at healthcare settings, is not there*” (IDI 5). In general, respondents agreed that, “*The government really has to do a lot in terms of advocacy, in terms of educating the entire population*” (FGD, HCP), and what is needed is to “*coordinate efforts in the mechanism on climate change response, encourage greening and green product activities and green growth economy, encourage transition from policy to action*” (FGD, Community Leader). According to many informants, the authorities must ensure climate change resilience and ensure, “*government investment in climate change in the urban space*” (IDI 3).

## 4. Discussion

There are major knowledge gaps on how to guide and develop interventions to improve climate change adaptation and resilience in relation to health at the local community level. A review of the literature from 2020 on health implications of climate change found just seven studies on climate change and health in LMICs [20]. The review identified studies reporting on food insecurity [21,22,23], water insecurity [22,23], illness [23,24,25], mental health [23], injuries from extreme weather events [22], and heat-exposure [26] related to climate change. All these risks were also reported by the participants in this study.

A knowledge, attitudes and practices (KAP) study in Guyana found that 36% of the 450 study participants experienced health hazards caused by climate change [17]. A study conducted in Tanzania among 399 participants from farming communities found that although 62% experienced the impact of changing weather on food production, the communities had very little knowledge on climate change and its impact on malaria and food security [27]. Conversely, a study among vulnerable communities in Bangladesh with 6720 participants found a clear perception about change in climate and related effects on health with 54% having knowledge on climate change [28]. Another study in Vietnam among residents in slum areas found 70% had heard about the impact of climate change on human health [29]. Almost all respondents in this study were aware of links between climate change and health, and experienced these in their lives. The higher awareness compared to previous studies was found with the caveat of this study being based on purposively sampled key informants and focus groups.

The findings of this study are limited to a specified area, group and setting. The number of participants interviewed was limited, which may introduce underestimation biases. Furthermore, the purposive sampling strategy may have introduced selection bias, which could lead to overestimating climate change related health risks. Thus, the findings of this study may not be generalizable at national and international levels. Furthermore, this study focused specifically on non-communicable diseases. Although participants also reported on communicable diseases, the interviews and sampling of participants were focused on NCDs, limiting the scope of this study.

The emerging themes of the IDIs and FGDs in this study were perceived disease risks related to climate change, climate change related risk factors for those diseases (nutrition and access to clean water, air pollution and waste management, lack of urban planning and public infrastructure, and economic factors), vulnerable groups, and potential adaptation strategies. Access to affordable and healthy food, which is at risk due to changing weather and deforestation, was a prominent sub-theme. Pollution from industries, in particular air and water pollution, was another important perceived health risk. Many participants identified that unplanned urbanization with dense settlements close to rivers without sufficient sewage systems and lack of public infrastructure, in particular toilets and affordable health care, exacerbated climate change related health risks. Respondents identified the poorest, children, the elderly, and those with NCDs as most at risk of climate change related health effects.

## 5. Conclusions

This study is the first to report on the experiences and perspectives of local health care workers and volunteers, informal and formal community leaders, and academia on climate change related health risks in an informal settlement in Africa. The seven main themes identified (climate change related diseases; nutrition and access to clean water; environmental risk factors; urban planning and public infrastructure; economic factors; vulnerable groups; and adaptation strategies) may guide further research and climate change resilience interventions, and enable information and advocacy campaigns to be tailored to the local context. This qualitative evidence on climate change related health risks offers important understandings which may be useful for urban planners, academia, health care workers and policy makers locally and nationally, in addition to those in the East Africa region. Interestingly, community leaders and health care volunteers in this study demanded more training and education about climate change and environmental health to protect themselves and their communities. Further research, including mixed-method approaches, covering the general inhabitants of informal settlements in Nairobi, in addition to other countries and regions, is warranted.

## Figures and Tables

**Table 1:** Results based on themes and sub-themes.

Themes	Sub-Themes	No. of Times Sub-Theme Was Mentioned
Climate change related diseases	Non-communicable diseases (NCDs) Infectious diseases Mental stress Injuries and accidents	81 mentions 36 mentions 6 mentions 3 mentions
Nutrition and access to clean water	Food insecurity Water insecurity Rising food prices Malnutrition and hunger	40 mentions 18 mentions 7 mentions 6 mentions
Environmental risk factors	Air pollution Waste management Contamination	41 mentions 26 mentions 20 mentions
Urban planning and public infrastructure	Sewage and drainage Housing Vegetation Infrastructure Health services Migration Urbanization	35 mentions 23 mentions 23 mentions 18 mentions 15 mentions 14 mentions 4 mentions
Economic risk factors	Poverty Lack of insurance against climate change hazards	28 mentions 9 mentions
Vulnerable groups	People living with NCDs (PLWNCD) and disabilities Children Elderly	11 mentions 9 mentions 3 mentions
Adaptation strategies	Education Policy Awareness	29 mentions 26 mentions 16 mentions

**Table 2:** Sub-themes represented across interviews (FGDs and IDIs).

	FGD Community Health Volunteers	FDG Health Care Professionals	FGD Community Leaders	IDI1 Academia	IDI2 Academia & Policy	IDI3 Academia	IDI4 Academia	IDI5 Administration & Policy
Climate change related diseases								
Non-communicable diseases (NCDs)	x	x	x	x	x	x	x	x
Infectious diseases	x	x	x	x	x	x	x	x
Mental stress	x		x		x	x		
Injuries and accidents			x					x
Nutrition and access to clean water								
Food insecurity	x	x	x	x	x	x	x	x
Water insecurity	x		x	x	x	x		x
Rising food prices	x	x			x	x		
Malnutrition and hunger	x	x	x	x				
Environmental risk factors								
Air pollution	x	x	x	x	x		x	x
Waste management	x	x	x			x	x	
Contamination	x	x	x					x
Urban planning and public infrastructure								
Sewage and drainage	x	x	x	x		x		x
Housing	x	x	x	x			x	x
Vegetation	x	x	x	x	x	x		x
Infrastructure	x	x	x		x	x	x	x
Health services				x	x	x	x	x
Migration	x		x	x	x			x
Urbanization	x	x		x				
Economic risk factors								
Poverty	x	x	x	x	x		x	x
Lack of insurance against climate change hazards	x	x				x		
Vulnerable groups								
People living with NCDs (PLWNCD) and disabilities	x		x			x	x	
Children	x	X	x		x			
Elderly		X	x					
Adaptation strategies								
Education	x	X	x	x	x	x	x	x
Policy		X	x			x	x	x
Awareness	x	X	x	x	x	x	x	

## Data Availability

Data supporting findings of the study are available upon reasonable request from the corresponding author (Johanne Greibe Andersen).
